# Response of different varieties of maize to nitrogen stress and diagnosis of leaf nitrogen using hyperspectral data

**DOI:** 10.1038/s41598-023-31887-z

**Published:** 2023-04-11

**Authors:** Yanli Lu, Xiaoyu Zhang, Yuezhi Cui, Yaru Chao, Guipei Song, Caie Nie, Lei Wang

**Affiliations:** 1 State Key Laboratory of Efficient Utilization of Arid and Semi-Arid Arable Land in Northern China/Key Laboratory of Plant Nutrition and Fertilizer, Ministry of Agriculture and Rural Affairs/ Institute of Agricultural Resources and Regional Planning, Chinese Academy of Agricultural Sciences, Beijing, 100081 China; 2 Inner Mongolia Agricultural University, Hohhot, 010018 China

**Keywords:** Plant sciences, Optical spectroscopy

## Abstract

Spectral technology is theoretically effective in diagnosing N stress in maize (*Zea mays* L.), but its application is affected by varietal differences. In this study, the responses to N stress, leaf N spectral diagnostic models and the differences between two maize varieties were analysed. The variety “Jiyu 5817” exhibited a greater response to different N stresses at the 12-leaf stage (V12), while “Zhengdan 958” displayed a greater response in the silking stage (R1). Correlation analysis showed that the spectral bands more sensitive to leaf N content were 548–556 nm and 706–721 nm at the V12 stage in “Jiyu 5817” and 760–1142 nm at the R1 stage in “Zhengdan 958”. An N spectral diagnostic model that considers the varietal effect improves the model fit and root mean square error (RMSE) with respect to the model without it by 10.6% and 29.2%, respectively. It was concluded that the V12 stage for “Jiyu 5817” and the R1 stage for “Zhengdan 958” were the best diagnostic stages and were more sensitive to N stress, which can further guide fertilization decision-making in precision fertilization.

## Introduction

Nitrogen (N) is one of the key factors in plant photosynthesis, ecosystem productivity, and leaf respiration. The low N use efficiency (NUE) of crops has always been a concern in N management, resource savings, and environmental protection. To obtain a high yield, farmers often apply an excessive amount of N fertilizer, which results in a low N utilization rate, severe losses of N fertilizer and environmental pollution. Soil analysis provide information of N availability, and N fertilizer requeriments (N rates) could be calculated based on nutrient grading method or target yield method^1^. Rational fertilization is based on the following main points. First, soil nutrient analysis determines the total amount of N fertilizer during the entire growth period, but the distribution of N fertilizer at different growth stages is typically based on farmer experience^2–4^. Second, determining the N status (abundance or deficiency) through plant nutrition diagnosis informs recommendations for fertilization rates according to the plant demand at that time^5^, and third, soil testing can be combined with plant nutrition diagnosis to determine the suitable N level more accurately^6^. Real-time monitoring of plant nutrient status and then supplying fertilizer on demand is a more effective way to improve fertilizer efficiency and optimize N regulation, which is an important aspect of precision fertilization. The amount and intensity of N demand by crops vary throughout the growth period. Timely and effective diagnosis of plant N status and determination of an accurate fertilization time are important prerequisites for achieving the synchronization of supply and demand and improving fertilizer utilization efficiency. Gebbers and Adamchuk^7^ proposed visible/near infrared (VIS/NIR) hyperspectral technology as a key technology for promoting precision agricultural development and solving food security problems. In precision agriculture, the development of hyperspectral technology has provided an effective method for fast, nondestructive, real-time monitoring of N status^8–12^. The spectral diagnosis of N status is based on the correlation between reflectance, which is sensitive to N, and the N concentration. Many spectral indices have been developed^13–16^, and the algorithm of the model has been continually optimized^17–20^. Partial least squares regression (PLSR), one of the optimization algorithms for modelling that combines the characteristics of principal component analysis, multiple linear regression analysis and canonical correlation analysis, uses data dimension reduction, information synthesis and screening technology and extracts new comprehensive components with the best interpretation ability of the system. It has been shown to be a powerful and popular method for multiple data analyses^21,22^.

Ground-based hyperspectral technology not only explains remote sensing data analysis on a large scale but also provides a way for farmers to predict N status in a timely manner and fertilize at the right time. Most previous studies have focused on monitoring the crop canopy to diagnose the chlorophyll or N status using spectral techniques at different scales^23–28^. However, canopy spectral data are mixed with information on the plant, soil, and other unknown information. Furthermore, by the time that canopy remote sensing reflects nitrogen stress, the plant in a serious nitrogen stress state, and the best time window for nitrogen supplementation is missed. Agriculture in China is characterized by a pattern of highly dispersed small-scale farm household management, and it is more practical to use the farmer’s field as a management unit^29,30^. The N concentration of the leaves, which is sensitive to spectra, is also affected directly by plant N status and should reflect any N deficiency. Leaf diagnosis can therefore provide more accurate information for fertilization decision-making.

There have been many studies determining the sensitive reproductive period for spectral diagnosis, determining the sensitive leaf, and optimizing the algorithms for diagnostic models^22,28,31^. There are many models of N nutrition based on spectral data in crops, and the visible bands sensitive to chlorophyll and NIR at 680–1100 nm have made considerable contributions to the models. However, the N diagnosis model is limited in its application; this limitation is partly due to the poor accuracy and universality of the model, which is affected by factors such as varietal differences^32,33^.

The physiological response of maize to N deficiency is always the basis of N diagnosis using spectral data. A clear understanding of the response of different crops or their cultivars to N stress is important for improving the precision of spectral diagnosis. There are differences in nitrogen uptake, nitrogen transport and nitrogen use efficiencies among different varieties. Additionally, differences in leaf structure such as leaf thickness, number of stomata and internal cell arrangement also affect the spectral reflectance^34,35^. The objective of this research is to clarify the differences in the responses of maize cultivars to different N fertilization rates, construct N diagnostic models and determine the optimal diagnostic time. This study provides a further basis for N stress diagnosis and the regulation of precision N fertilization under the current pattern of farm management in China.

## Materials and methods

### Experimental field

The experiments were conducted from 2019 to 2020. The experimental field was located in the international agricultural high technology industry park of the Chinese Academy of Agricultural Sciences, Wanzhuang Town, Langfang City, Hebei Province (116° 35′ 16′ E, 39° 35′ 47′ N), China, with a temperate continental climate and an annual mean temperature of 11.9 °C. The soil type was fluvo-aquic with a sandy soil texture, and the cropping system was a long rotation of summer maize and winter wheat with deep ploughing and harrowing before sowing each season. The soil samples from the cultivated layer (0–20 cm) were collected for chemical analysis before sowing, and the basic physicochemical properties of the test soil are shown in Table 1.

**Table 1:** Soil nutrient properties of the experimental field.

Year	pH	Organic matter (g/kg)	Nitrate nitrogen (mg/kg)	Ammonium nitrogen (mg/kg)	Available phosphorus (mg/kg)	Available potassium (g/kg)
2019	7.75	10.40	25.80	17.90	21.90	69.90
2020	8.07	10.70	33.70	13.10	23.50	51.40

### Experimental design

Four N application rates (0, 60, 120, and 180 kg/hm^2^, denoted N0, N1, N2, and N3, respectively) with three replicates and 24 experimental plots with an area of 32 m^2^ each (4 m × 8 m) were arranged in a randomized block design. As recommended by an efficient soil nutrient determination method proposed by A. H. Hunter of the Agro.Service International Inc.in Florida, United States (abbreviated as ASI method )^36^, the optimum amounts of fertilization were 180 kg/hm^2^ nitrogen (N), 90 kg/hm^2^ phosphorus (P_2_O_5_) and 60 kg/hm^2^ potassium (K_2_O); the phosphorus and potassium were applied at the seedling stage as base fertilizers. Two maize (*Zea mays* L.) varieties, “Jiyu 5817” and “Zhengdan 958”, were selected for this experiment; both are popular varieties in North China. “Zhengdan 958” is a locally dominant variety that has been planted for 12 years at the test site. There are differences in nitrogen uptake, nitrogen transport and nitrogen use efficiency as well as plant morphology, leaf colour, and internal structure of the leaf between “Zhengdan 958” and “Jiyu 5817”^35,37^. The planting density was 66,667 plants/hm^2^ for each variety, and weeds and pesticides were sprayed in a timely manner during the growth period. The main characteristics of the two maize varieties are shown in Table 2.

**Table 2:** Main characteristics of the tested maize varieties.

Variety	Plant height (cm)	Ear position height (cm)	Lines per ear	Growth days	Crude protein (%)	Crude starch (%)	Crude fat (%)	Lysine (%)
JY5817	260	105	16	103	8.68	79.93	4.11	0.28
ZD958	240	100	15	96	8.47	73.42	3.92	0.37

### Sampling and yield determination

The sampling periods were the 6-leaf stage (V6), 12-leaf stage (V12), flowering and silking stage (R1), filling stage (R2), waxing stage (R5) and ripening stage (R6). At the seedling stage, plants with the same growth trend were tagged and used for sampling at the next few stages. Before the VT stage (tasselling stage), the top fully expanded leaves of the maize plants were collected, and after the VT stage, the ear leaves were taken, which are collectively referred to as key functional leaves. The yield of different treatments was calculated based on the weight of grain harvested in each plot.

### Determination of nitrogen content and accumulated nitrogen in maize leaves

The samples in paper bags were placed in an oven at 105 °C for 30 min for enzyme fixation; the temperature was then set to 70 °C, and drying was continued until a constant weight was reached. The dried samples were ground and passed through a 60-mesh screen, digested using the H_2_SO_4_–H_2_O_2_ method, and finally assessed with an AA3 flow injection analyser (SEAL Analytical GmbH, Norderstedt, Germany). Leaf N accumulation was calculated as the leaf N content multiplied by the leaf dry weight.

### Measurement of spectral reflectance

The spectra of different leaves were measured with an ASD FieldSpec 3 spectrometer with a high-intensity contact probe (PANalytical, B. V, Boulder, Colorado, USA; formerly Analytical Spectral Devices). After the samples were collected, the spectra were detected with the high-intensity contact probe by clamping the leaf and avoiding the veins. Five positions from the tip to the base of each leaf were measured, and 10 internal scans were made for each measurement. The 10 spectra were then averaged into one spectrum to represent the spectrum of that position; the average of the 5 positions was calculated for each leaf, and finally, the average of the leaves was calculated for a sample.

### Data processing, model calibration and validation

Spectral data preprocessing was performed using Viewspectpro 5.7 software and Microsoft Excel, and an ANOVA was performed using IBM SPSS statistical software. Multivariate data analysis software, Unscrambler 9.7, was used to construct and validate the model, which was constructed using partial least squares (PLS1) regression and cross-validation. In Unscrambler, models are tested using external samples collected by the same method as that of the calibration set. A total of 25 external samples were used for the “Jiyu 5817” and “Zhengdan 958” models alone, and a total of 50 samples were used to test the integrated model. In this paper, we tested the model in three ways. First, a model of one variety, i.e., 25 external samples of “Jiyu 5817”, was used to test the model constructed for “Jiyu 5817”. Second, a model for the second variety, i.e., the “Zhengdan 958” samples, was used to test the model constructed for “Jiyu 5817”; next, the “Jiyu 5817” samples were used to test the model constructed for “Zhengdan 958”. Third, an integrated model was tested using different samples from different varieties, i.e., samples of “Jiyu 5817” or “Zhengdan 958” were used to test the integrated model.

### Statement of compliance

“Zhengdan 958”, the maize (*Z*. *mays* L.) cultivar used in the present experiment, complied with international guidelines. We complied with the IUCN Policy Statement on Research Involving Species at Risk of Extinction and the Convention on the International Trade in Endangered Species of Wild Fauna and Flora.

## Results

### Changes in the N content and N accumulation in key functional leaves of maize under different N application rates

**Figure 1:**
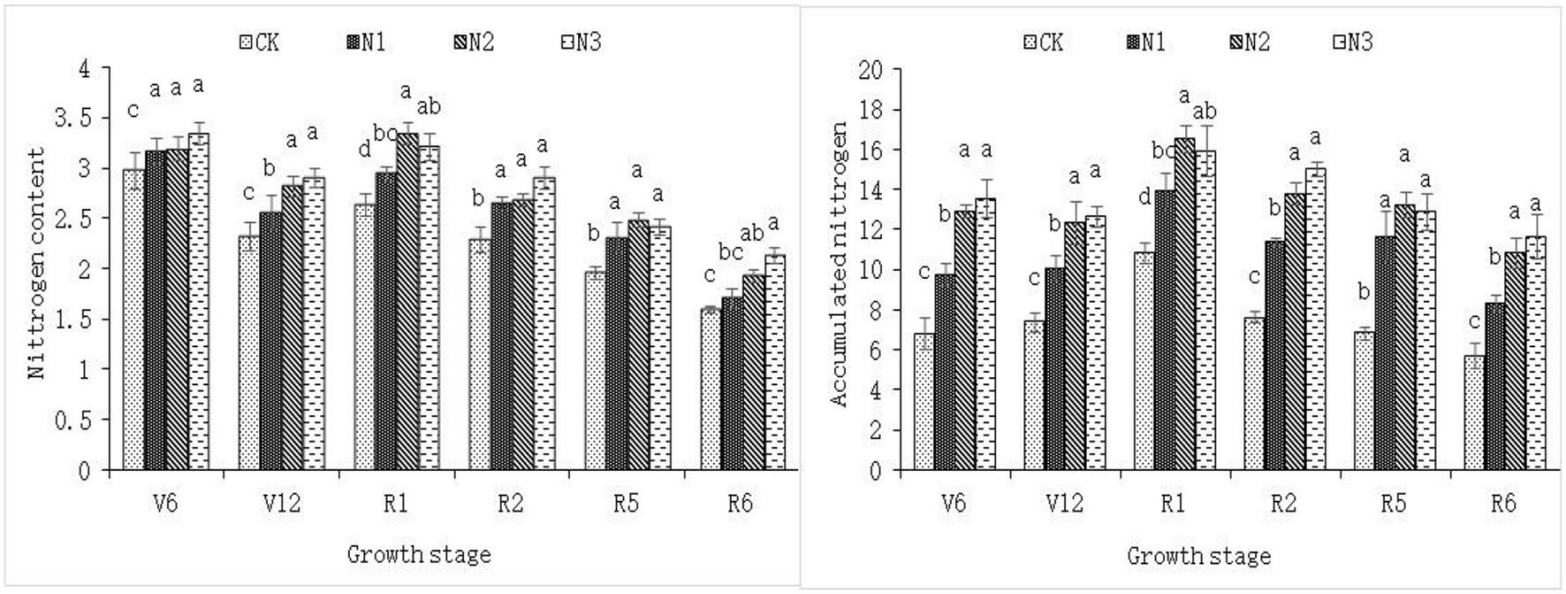
N content and accumulation in the key functional leaves of “Jiyu 5817” maize under different N rates. CK, N1, N2 and N3 represent N rates of 0, 60, 120 and 180 kg/hm^2^, respectively.

Figure 1 shows the changes in the N content and N accumulation in key functional leaves of “Jiyu 5817” during the growth period. Overall, as the N application rate decreased, both metrics showed a decreasing trend with the progression of the growth period. There was no significant difference in the N content or N accumulation in key functional leaves when N was applied at 120 kg/hm^2^ or 180 kg/hm^2^ (N2 and N3 treatments), and the recommended N application rate of 180 kg/hm^2^ (N3 treatment) resulted in the highest N content and N accumulation at the ripening stage (R6). When the N application rate was less than 120 kg/hm^2^, N accumulation decreased significantly with decreasing N application. Both the N content and N accumulation showed significant variation among the different treatments at the V12 stage, which is also the most important N requirement period and the key period for topdressing.

**Figure 2:**
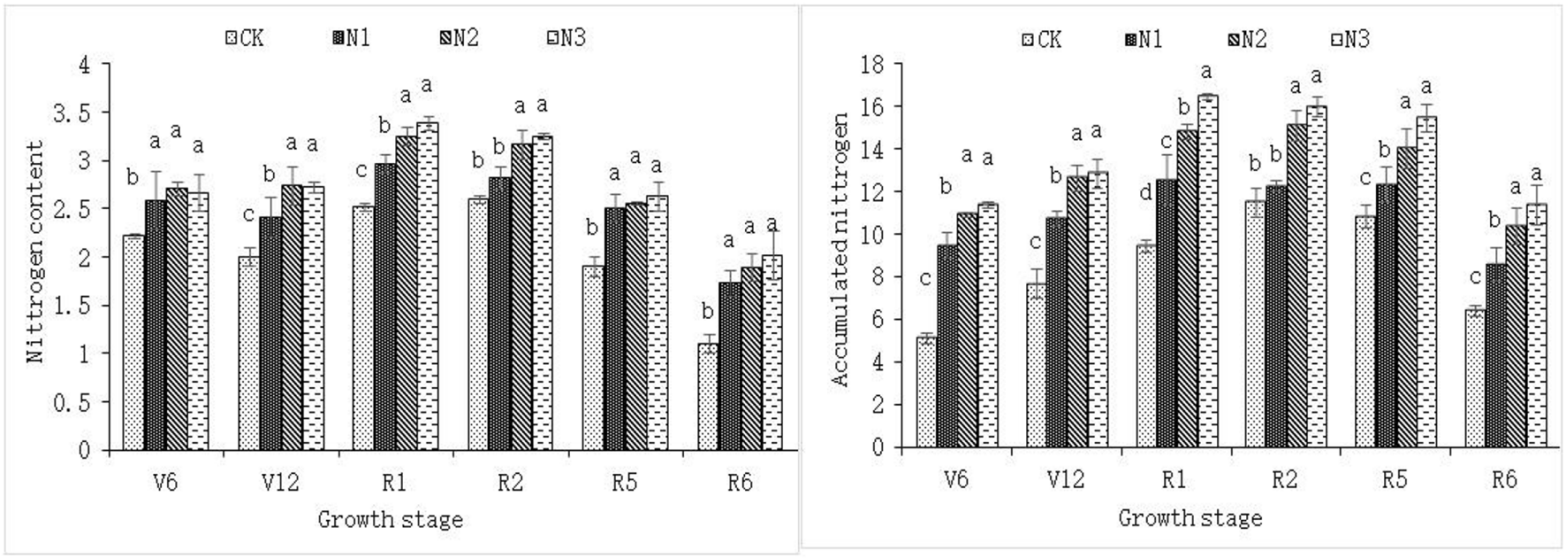
N content and accumulation in the key functional leaves of “Zhengdan 958” maize under different N rates. CK, N1, N2 and N3 represent N rates of 0, 60, 120 and 180 kg/hm^2^, respectively.

Figure 2 shows the changes in the N content and N accumulation in key functional leaves of “Zhengdan 958” during the growth period, and the overall change trend was consistent with that of “Jiyu 5817”. There was also no significant difference in the N content in key functional leaves between the N2 and N3 treatments, but the difference in N accumulation was significant among the different N treatments at the silking stage (R1). At the ripening stage (R6), the recommended N application rate of 180 kg/hm^2^ (N3) resulted in the highest N content and N accumulation. At the R5–R6 stage, except for the group without N application (N0), there was no significant difference in the N content among the other three treatment groups, and there was no significant difference in leaf N accumulation between N3 and N2; however, when the N application rate was less than 120 kg/hm^2^, the N accumulation decreased significantly with increasing N stress. Overall, considering both the N content and N accumulation, the period of “Zhengdan 958” sensitivity to N stress was from the V12 to R1 stages, of which R1 was the most informative because the N accumulation during this stage was the most sensitive to N stress.

### Yield analysis of different maize varieties

As shown in Table 3, the yield was relatively sensitive to different N stresses; N deficiency significantly reduced the yield, and the average yields of “Jiyu 5817” and “Zhengdan 958” reached their maximum values under the N3 treatment. Compared with that under the N3 treatment, the average yields of “Jiyu 5817” under the N0, N1 and N2 treatments were decreased by 24.04%, 7.76% and 3.11%, respectively, but the N2 treatment was not significantly different from the N3 treatment. The average yields of “Zhengdan 958” under the N0, N1 and N2 treatments decreased by 22.26%, 13.91% and 7.66%, respectively, and the average yield showed a significant difference under the different N treatments. Based on the yield response to the different N application rates, 180 kg/hm^2^ (N3) was recommended as the optimum N application rate for the two maize varieties to achieve the highest yield. The changes in the yields of “Jiyu 5817” and “Zhengdan 958” were basically consistent with the change in the N in the ear leaf, particularly at maturity. In “Zhengdan 958”, there was no difference in the N content or accumulation between N2 and N3, but the yield under N3 was significantly higher than that under N2; in “Jiyu 5817”, there were no significant differences between N2 and N3 in the N content, N accumulation or yield.

**Table 3:** Yields of “Jiyu 5817” and “Zhengdan 958” under different N rates.

N rate (kg/hm^2^)	JY5817 (kg/hm^2^)	Standard error	ZD958 (kg/hm^2^)	Standard error
0	11,397 c	268	12,002 d	151
60	13,839 b	260	13,290 c	288
120	14,536 ab	298	14,255 b	321
180	15,003 a	420	15,439 a	197

Different lowercase letters in the table indicate a significant difference of 0.05 (*p* < 0.05).

### Correlations between N accumulation and spectral reflectance in key functional leaves of different maize varieties

**Figure 3:**
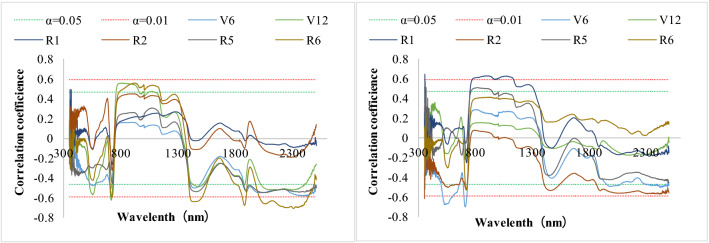
Correlation coefficients between N accumulation and spectral reflectance in the key functional leaves of maize (“Jiyu 5817”, left and “Zhengdan 958”, right).

The correlation between N accumulation and spectral reflectance in key functional leaves of different maize varieties was analysed. Figure 3 (left) shows that the correlation between N accumulation and spectral reflectance in key functional leaves of “Jiyu 5817” was significant from 532 to 565 nm, 700 to 716 nm and 1406 to 1485 nm at the 6-leaf stage (V6) and from 525 to 576 nm, 706 to 721 nm, 756 to 955 nm, 1421 to 1506 nm and 2018–2398 nm at the 12-leaf stage (V12). There was a significant correlation from 705 to 733 nm and 785 to 1138 nm and a significant correlation from 1397 to 1519 nm, 1848 to 1889 nm and 2000 to 2430 nm at the ripening stage (R6). Figure 3 (right) shows the correlation between N accumulation and spectral reflectance in key functional leaves of “Zhengdan 958”. At the V6 stage, there was a significant negative correlation from 508 to 724 nm and 1972 to 2100 nm and an extremely significant correlation from 509 to 597 nm and 697 to 724 nm. A significant negative correlation was observed from 760 to 142 nm at the silking stage (R1) and from 712 to 724 nm at the ripening stage.

### Establishment of prediction models of maize leaf N content based on PLSR

**Figure 4:**
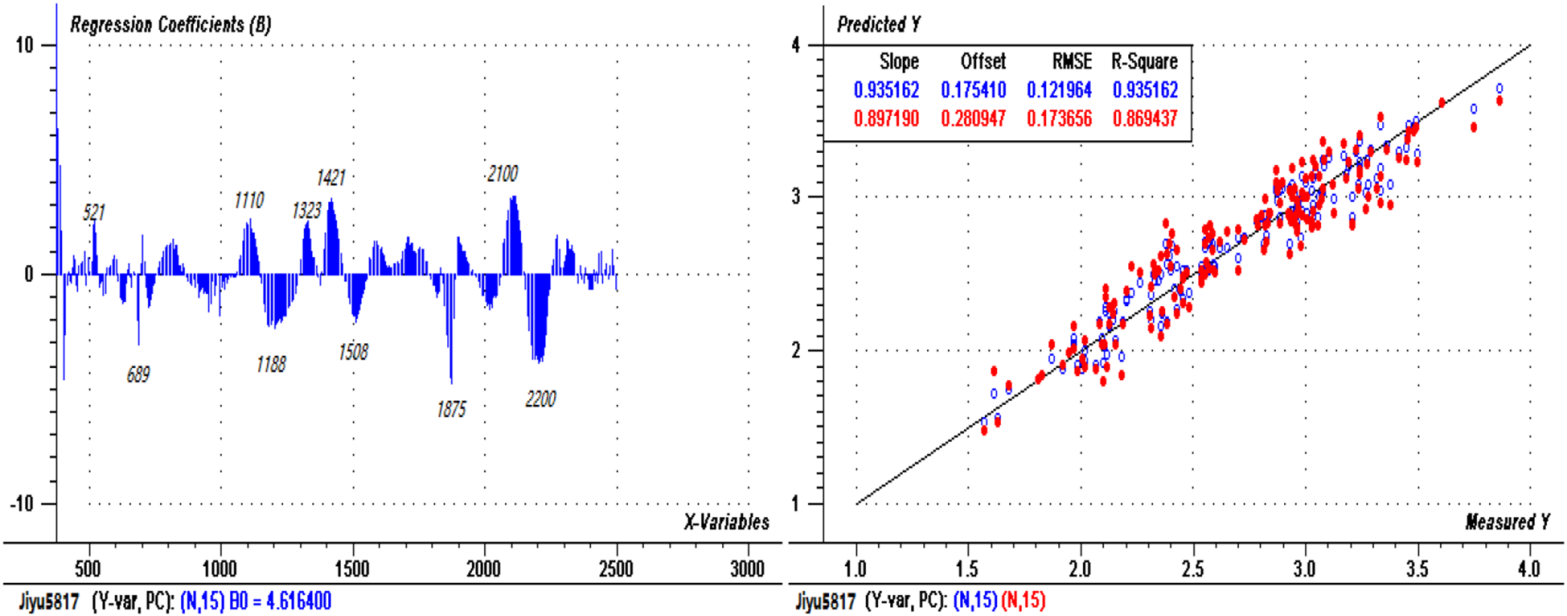
PLSR regression coefficients (left) and prediction evaluation diagram (right) for “Jiyu 5817”.

**Figure 5:**
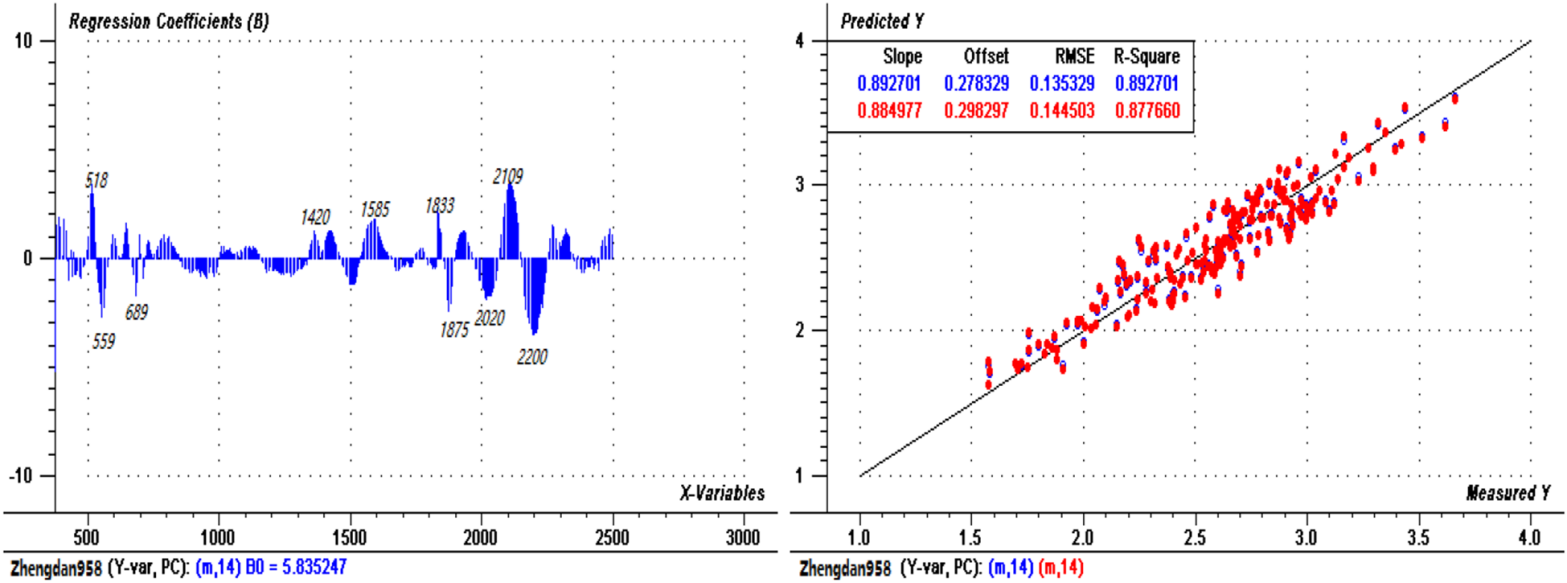
PLSR regression coefficients (left) and prediction evaluation diagram (right) for “Zhengdan 958”.

The N spectral prediction models of the two varieties were established using the PLSR method. Figures 4 and 5 show the regression coefficient diagrams (left) and prediction evaluation diagrams (right) of the models for “Jiyu 5817” (sample size n = 165) and “Zhengdan 958” (sample size n = 216), respectively. For “Jiyu 5817” (Fig. 4), the root mean square error (RMSE) and the coefficient of determination (R^2^) of the calibration set were 0.122 and 0.935, respectively, and the RMSE and R^2^ of the validation set were 0.174 and 0.860, respectively; the top 10 central wavelengths that contributed to the model were 521 nm, 689 nm, 1110 nm, 1188 nm, 1323 nm, 1421 nm, 1508 nm, 1875 nm, 2100 nm and 2200 nm. For “Zhengdan 958” (Fig. 5), the RMSE and R^2^ of the calibration set were 0.135 and 0.883, respectively, and the RMSE and R^2^ of the validation set were 0.145 and 0.878, respectively; the top 10 central wavelengths that contributed to the model were 518 nm, 559 nm, 689 nm, 1420 nm, 1585 nm, 1833 nm, 1875 nm, 2020 nm, 2109 nm and 2200 nm.

**Figure 6:**
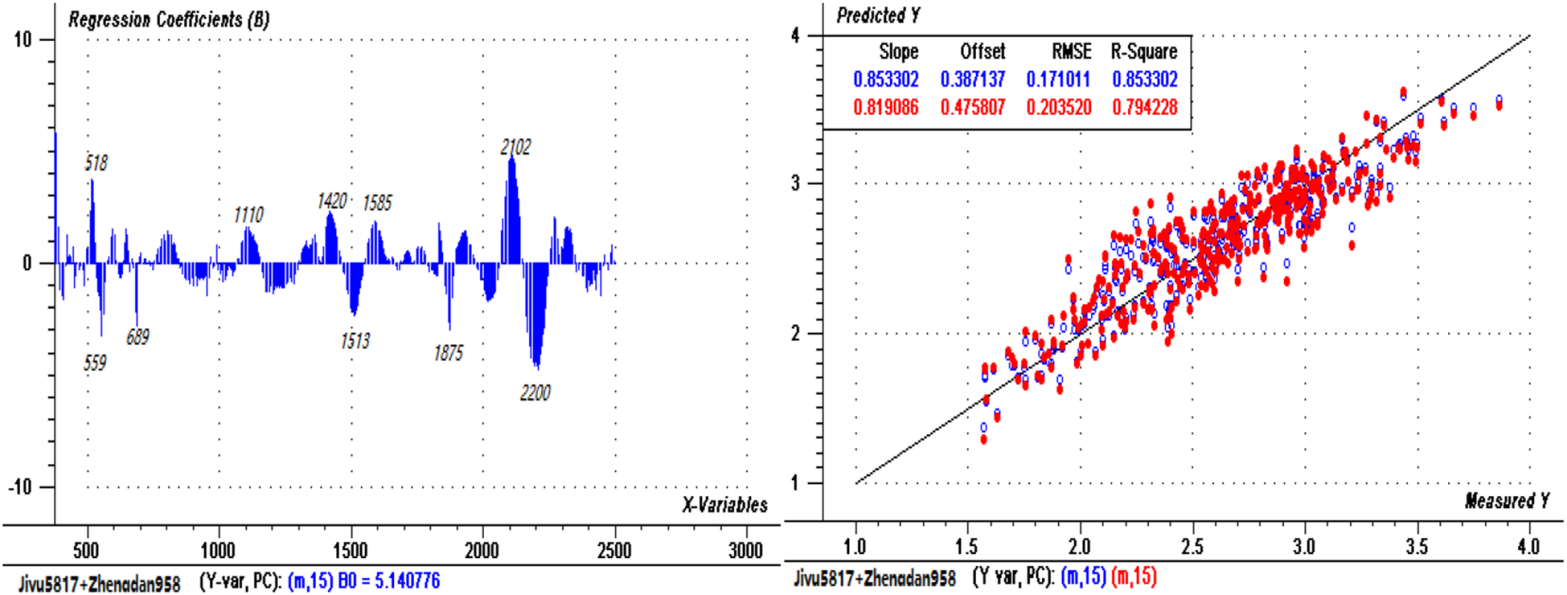
PLSR regression coefficients (left) and prediction evaluation chart (right) of the combined maize varieties.

The data from the two varieties were combined for further analysis. Figure 6 shows the regression coefficient diagram and prediction evaluation diagram of the spectral prediction model of leaf N content in the key functional leaves of the two maize varieties (sample size n = 381). The RMSE of the validation set model was 0.204, the R^2^ was 0.794, and the number of principal components was 15. The top 10 central wavelengths were 518 nm, 559 nm, 689 nm, 1110 nm, 1420 nm, 1513 nm, 1585 nm, 1875 nm, 2103 nm and 2200 nm.

Figures 4, 5 and 6 show that most of the positions of the central bands with greater contributions were essentially similar in the three models. Compared with that of the integrated model of the two varieties (not considering the varietal differences), the prediction accuracy of the model with variety classification was improved; the accuracies of the calibration and validation sets of the combined varieties were lower than those of the individual varieties, but the combined model was also validated as statistically acceptable. Therefore, to simplify the prediction of leaf N content, a general model can be applied for different varieties. However, due to the different responses to N stress by the two varieties, the effects of varietal differences should be considered in the application of the model.

### External test of the prediction models

As shown in Table 4, the determination coefficient (R^2^) of all the evaluation results was greater than 0.803 and the relative error was less than 8.98%. The order of model test accuracy from high to low was the model of the same variety, the integrated model validation and the model of the opposite variety. Although the determination coefficients and errors of the prediction model and its validation results were not particularly desirable, as a rapid, real-time, and nondestructive N nutrition diagnostic technique that can be applied under field conditions, it is sufficient to provide a reference basis for N regulation and management during the growing period of maize.

**Table 4:** Test results of the prediction model based on external samples.

Number	Sample source for testing	Tested model	Sample number	Coefficient of determination (R^2^)	Average relative error (RE %)
1	Jiyu 5817	M1	25	0.873	5.63
2	Zhengdan 958	M2	25	0.861	6.21
3	Jiyu5817 + Zhengdan 958	M3	50	0.856	6.74
4	Jiyu5817	M2	25	0.821	8.01
5	Zhengdan958	M1	25	0.803	8.98

M1 represents the PLSR prediction model of “Jiyu 5817”, M2 represents the PLSR prediction model of “Zhengdan 958”, and M3 represents the PLSR prediction model of the two varieties combined. For example, Line 1 shows that Model 1 (M1) is tested using 25 unknown samples of “Jiyu 5817”, whereas Line 4 shows that Model 2 (M2) is tested using 25 unknown samples of “Jiyu 5817” (i.e., samples from Jiyu 5817 were used to test the Zhengdan 958 model.) The explanations for the other lines are similar.

## Discussion

Monitoring the nitrogen (N) status of plants using spectral technology is the premise of precision fertilization, which is part of precision agriculture. Excessive N fertilizer input has many disadvantages such as wasted resources, environmental pollution and the destruction of the soil structure. The Chinese government is paying increasing attention to the efficient utilization of fertilizer resources and the pollution caused by chemical fertilizers. In 2015, the Ministry of Agriculture proposed the "zero increase action plan for chemical fertilizers and pesticides". To ensure food security and environmental friendliness, it is imperative to optimize the fertilizer utilization rate. Timely and effective diagnosis of the N nutritional status plays an important role in optimizing N management and improving utilization efficiency in precision fertilization. With the development of spectral technology, an increasing number of people are using spectrometers such as SPAD, GreenSeeker, and hyperspectral instruments to monitor crop N nutrition^38–40^, even in the field of crop breeding as a reference for varietal screening^41,42^. Regarding the spectral diagnosis of N nutrition, researchers have always made efforts to improve the diagnostic accuracy from various perspectives^26,28,31,41,43^.

The application of spectral technology in diagnosing maize N nutrition is still affected by various factors, which has limited its popular use. Regardless of the type of diagnostic technology, the physiological response of the plant/leaf to N abundance and deficiency is the basis of spectral diagnosis and cannot be ignored. N is a highly mobile element in plants, and its distribution in plants takes the form of a gradient. Ciganda et al.^44^ concluded that a bell-shaped curve provided a very good fit for the vertical distribution of chlorophyll content regardless of the crop growth stage. In theory, in the early stage of crop growth, because N is preferentially supplied to the newest leaves, the response of plants to N stress is to optimize photosynthesis by changing the vertical distribution of N to prevent the upper leaves from being stressed^28^. After entering the reproductive growth stage, the emphasis is on promoting N transport to support grain filling, and the change in the N dynamics in ear leaves is particularly important. Compared with canopy spectral monitoring, leaf diagnosis also avoids differences in variety appearance and growth to a certain extent^8,45^. In this study, the key functional leaf (the top fully expanded leaf before VT and the ear leaf after VT) was taken as the diagnostic target.

There is still no clear answer regarding whether varietal differences are the dominant influencing factor in the spectral diagnosis of N nutrition in maize. Zhou et al.^32^ noted that varietal differences should be considered in the research and production application of hyperspectral technology to N nutrition diagnosis in maize. Regarding differences in cropping systems, Wen et al.^11^ found that the best 2-band VIs and the PLS regression based on selected FDR wavelengths provided a useful exploratory tool for estimating the leaf nitrogen concentration (LNC) of maize across years, ecological areas, and asynchronous growth stages. Lu et al.^11^ proposed an N stress spectral index (NSSI) based on the ratio of the target treatment to the N-sufficient treatment, which can eliminate the influence of factors such as variety, growth period and environmental conditions. In this paper, we compared the differences between two maize varieties in response to N stress and evaluated their N prediction models.

The study of varietal differences provides a basis for solving the problem of whether to consider variety as a factor in the application of the model. Based on the physiological mechanism and the spectral response of N abundance and deficiency in maize, the similarities and differences in the diagnosis of N nutrition in different varieties via spectral analysis were clarified; specifically, the N content and N accumulation in maize leaves were insufficient under N stress, and the yield was significantly affected. The response of the N content of the key functional leaves to different N levels varies with the growth period, and the determination of sensitive stages provides a basis for the spectral diagnosis of N nutrition. As shown in this paper, there is little difference between the combined model and the individual models of the two varieties, so the spectral diagnostic model of leaf N content does not take the variety factor into account. However, in practical applications, different varieties have different periods of sensitivity to different N stresses; therefore, to ensure an accurate diagnosis, the most sensitive period should be selected when using the model to diagnose N stress. This study provides a basis for solving the problem of whether to consider the variety factor in the application of the model. Further research will focus on combining the phenotypic and physiological characteristics of varieties, soil fertility, and the relationship to canopy spectra to improve the diagnostic accuracy and realize precision variable fertilization on farmers’ fields.

## Conclusions

The recommended N application rate (N3) maintained a high N content and N accumulation during the entire growth period and achieved the highest yield. V12 and R1 were determined to be the stages most sensitive to N stress in “Jiyu 5817” and “Zhengdan 958”, respectively, and spectral reflectance from 548 to 556 nm and 706 to 721 nm for “Jiyu 5817” and from 760 to 1142 nm for “Zhengdan 958” were also most sensitive to the leaf N content at the V12 and R1 stages, which provides a basis for further diagnosis using spectral data. The sensitive period of each variety in response to N application rates should be considered in diagnosing N stress. Compared with the model established by combining the two varieties, the model established by individual varieties produced a higher determination coefficient (R^2^) and lower root mean square error (RMSE); R^2^ increased by 10.6% and RMSE decreased by 29.2%, and the best spectral diagnostic stages were the V12 stage for “Jiyu 5817” and the R1 stage for “Zhengdan 958”.

## Data Availability

The datasets used and/or analysed during the current study are available from the corresponding author on reasonable request. Correspondence and requests for materials should be addressed to LU Yanli or Wang Lei.
